# Common eye conditions in children: care and referral at primary level

**Published:** 2024-02-09

**Authors:** Sucheta Kulkarni, Dupe Ademola-Popoola

**Affiliations:** 1 Senior Consultant: PBMA's H. V. Desai Eye Care Institute, Department of Ophthalmology, Pune, India; 2 Professor of Ophthalmology: Paediatric, Strabismus and Ophthalmic Oncology, University of Ilorin& University of Ilorin Teaching Hospital, Ilorin, Nigeria.

**Believe parents and caregivers when they say there is something wrong with their child's eye(s) or vision. As some conditions are blinding if not diagnosed and managed promptly, refer the child if you are in any doubt.**

**Figure:**
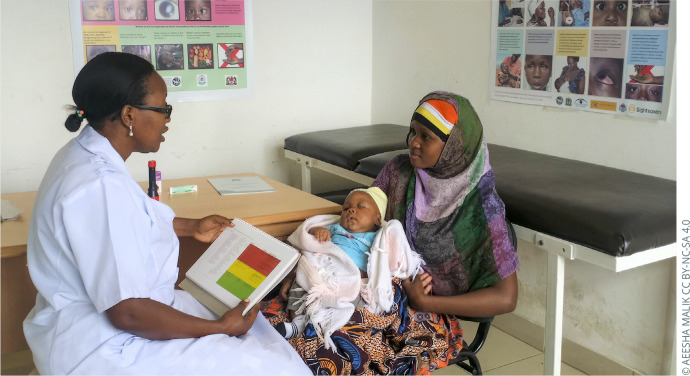
Believe parents when they say there is something wrong with their child's eyes. tanzania

When a child presents with an eye problem, it is important to listen carefully to what the parent or caregiver says about what they have noticed, and to believe them when they say there is something wrong with their child's eyes or vision – they are nearly always right.

In this article, we discuss the most common eye conditions in children. These can be managed at community or primary level by either treating the child, or referring them to the nearest hospital with an ophthalmologist. If you need to refer a child, explain the level of urgency to the parent or caregiver and check that they understand where to go and when they must be there.

One of the first things you will notice about a child is their age; this is an important clue when finding out what might be wrong with their eyes. We have therefore listed the common eye conditions by the age they most commonly occur, starting with newborns and ending with older children and adolescents.

## A newborn baby with profuse discharge

During birth, a baby's eyes can be infected by organisms present in the birth canal. Two organisms that have the most severe consequences for the baby, and therefore require urgent treatment, are *Neisseria gonorrhoea* (the organism which causes gonorrhoea) and *Chlamydia trachomatis* (responsible for chlamydia).

**Figure 1:**
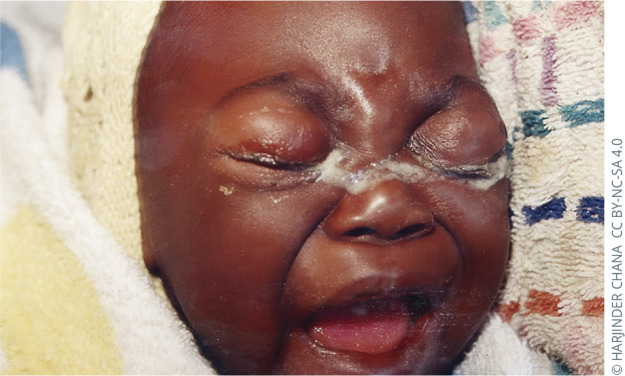
A baby with profuse discharge due to gonococcal infection. zimbabwe

If the mother has gonorrhoea, there is a high likelihood that the newborn baby will develop very severe bilateral conjunctivitis within 3–5 days of birth, with swollen eyelids and profuse, thick, yellow discharge (Figure 1). The condition can lead to corneal abscesses and blindness.

If the mother has chlamydia, there is a high likelihood that the baby will develop swollen eyelids and discharge within 5–14 days after birth. If untreated, it may cause corneal scarring as well as lung infections.

### What to do

- If you suspect a newborn has one of these conditions, **refer them urgently (on the same day if possible)** to a hospital where there is an ophthalmologist.
- Explain to parents the seriousness of the condition, i.e., the risk to their baby's sight and health. Explain the dangers of traditional treatments and the importance of getting to the hospital without delay (ideally on the same day).
- Wash your hands thoroughly after examining these babies, as gonorrhoea is highly infectious, and it's not possible to be sure which condition is responsible for the discharge you are seeing.

### What care is needed?

At the hospital, investigations will be done to determine which organism is responsible, so the correct antibiotic can be given.

If **gonorrhoea** is confirmed, the recommended treatment is ceftriaxone as a single dose of 25–50 mg/kg intramuscular or intravenous, up to a maximum of 125 mg. Note: penicillin is no longer recommended, as the bacteria responsible has become resistant to penicillin in many countries.

If **chlamydia** is confirmed, the recommended treatment is oral azithromycin 20mg/kg once daily for three days, or oral erythromycin 50 mg/kg/day, divided into 4 doses, for 14 days.

Parents and their sexual partners will require treatment if they have either of these conditions.

## Newborns and babies with watering eyes

Two very different conditions can cause watering eyes in newborn and young babies whose eyes are **not red**: one is a **blocked tear duct**, which usually resolves after a few months, and the other is **congenital glaucoma**, which can lead to blindness if not managed correctly.

If the ‘whites’ of the eyes are white, the watering eyes are likely due to a **blocked tear duct** when:

- Both eyes are a normal size
- Both corneas are clear and bright
- The baby is not distressed or in pain
- The baby does not object to a bright light.

**Figure 2:**
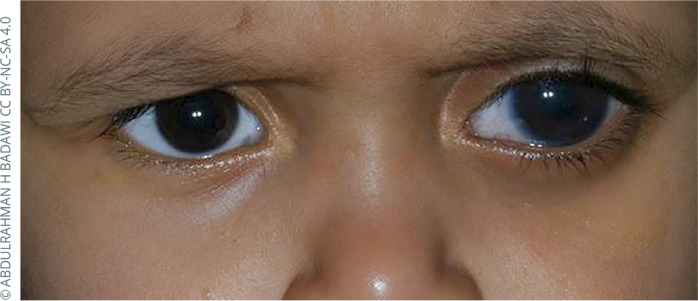
This child has congenital glaucoma in the left eye, which is larger than the right eye. saudi arabia

If the ‘whites’ of the eyes are white, the watering eyes are likely due to **congenital glaucoma** (Figure 2) when:

- One or both eyes are larger than normal (known as buphthalmos)
- The cornea(s) are hazy or cloudy (although this may not be visible)
- The child objects to a bright light and seems to be in pain.

### What to do

If you suspect a **blocked tear duct**:

- Reassure the parents that it is likely to get better.
- Suggest that parents try tear duct massage, a common treatment for a blocked tear duct: press a clean fingertip against the inner corner of the eye (nearest the nose) and massage downwards, towards the tip of the nose. Parents can do this several times a day, which may help to clear the blockage.
- Tell parents to bring their child back at the age of 9 months if the eye is still watering.
- If the tear duct is still blocked at 9 months, refer the child for a minor procedure to unblock the duct.

If you suspect **congenital glaucoma**:

- Refer the child **urgently** (within 1 week) to a hospital with an ophthalmologist specialising in child eye care.
- Explain to the parents or carers that, the sooner the child is treated, the better the results will be.

### What care is needed

Read the article on congenital glaucoma in this issue for more information on the diagnosis, treatment, and long-term management of this condition.

## A baby or child with something white inside the eye

The parent or carer may say they have noticed “something white” or a “reflection” in one or both eyes of the child. Mothers often notice this while breastfeeding.

Ask the mother whether there is a family history of **childhood cataract** (opacity of the lens) or **retinoblastoma** (a malignant tumour of the retina), as these are the two most important causes of a white reflex (also known as leukocoria) in children.

Another cause of a white reflex, if the baby was born prematurely, is **retinopathy of prematurity** (ROP), a condition that is preventable but potentially blinding. Premature babies must be examined by an ophthalmologist within the first month of life, as blindness can develop rapidly. Refer the child if needed. Visit www.cehjournal.org to read our 2017 issue on this topic.

### What to do

**Figure 5:**
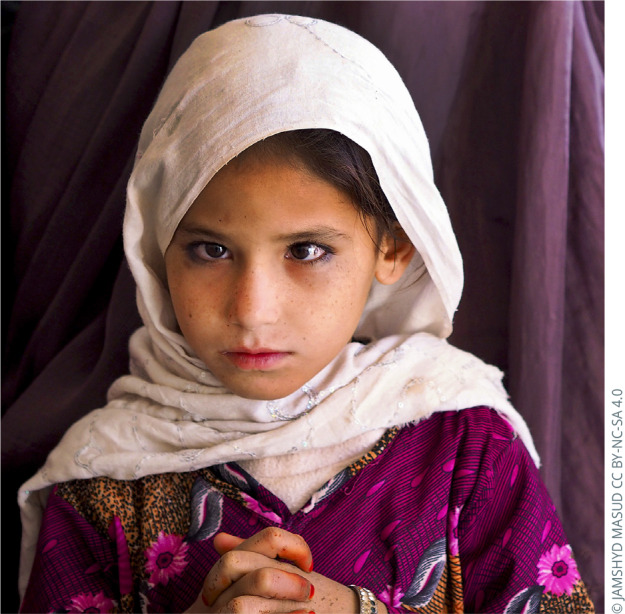
Child with a squint; the left eye is turning in. pakistan

- Examine the eyes to see if they are straight or whether a squint is present (see Figure 5).
- If you have an indirect ophthalmoscope or Arclight, try to elicit the fundal (red) reflex.
- Even if you cannot see anything wrong, it is always advisable to believe what the mother has said.
- **Refer the child urgently to an ophthalmologist**, even if you cannot see anything wrong and there is no family history. Explain to the carers that it is very important that the child is examined by an eye care specialist as soon as possible (in less than 1 week).

### What care is needed

**Figure 3:**
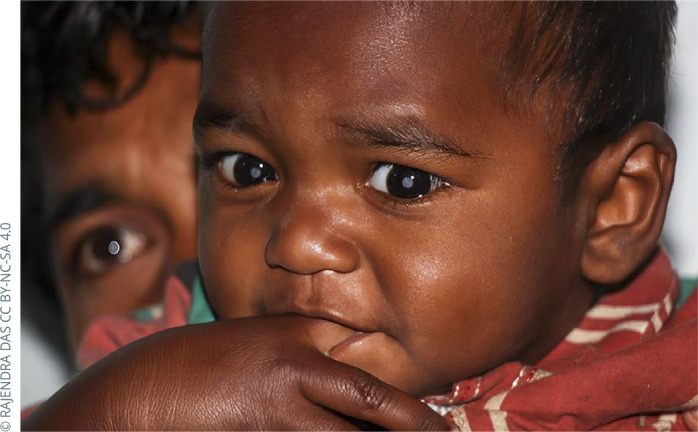
White pupils in both eyes, due to cataract. india

**Figure 4:**
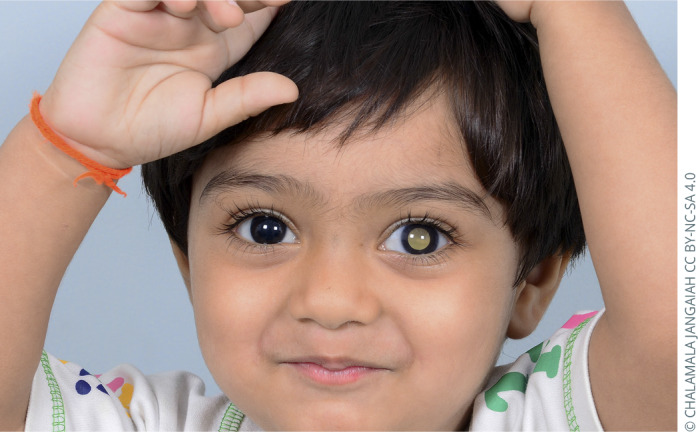
A child with retinoblastoma in the left eye. india

- A child with **cataract** (Figure 3) must undergo surgery to remove the cataract as soon as possible, so that light can reach the retina and the brain can develop normally. If the cataract is removed too late, the child may not be able to see with that eye, as the necessary brain development did not take place. See the article about congenital cataract in this issue.
- A child with **retinoblastoma** (Figure 4) must receive urgent treatment to prevent the cancer from spreading further, which can result in the death of the child. Visit www.cehjournal.org to read the *Community Eye Health Journal* issue on retinoblastoma, published in 2018.

## A baby or child with a painful red eye (or eyes)

If a baby or child presents with one or both eyes that are red, painful, and light sensitive, they may have a corneal ulcer. Gently open the eye and examine it using a torch or an Arclight ophthalmoscope. If you can see a cloudy patch on the surface of the cornea, this is likely to be a corneal ulcer. If the ulcers are the result of measles infection and/or vitamin A deficiency, both eyes will be affected.

Corneal ulcers can result in corneal scars and blindness if they are not treated in time.

### What to do

- If you suspect vitamin A deficiency, give the child a high dose of vitamin A (100,000 IU if aged 6–12 months and 200,000 IU if aged 6–59 months).
- Even if you cannot clearly see a corneal ulcer, refer the child **very urgently (ideally within 1 day)** to the nearest hospital where there is an ophthalmologist.
- Explain to parents the seriousness of the condition and the need for their child to be seen without delay.
- Explain the dangers of traditional treatments or remedies.

### What care is needed

- The child needs to undergo urgent investigation to determine the cause of the ulcer so the correct treatment can be given.

## Parents say their child cannot see properly

If a parent or caregiver thinks their child cannot see properly, find out why they think this is so.

- **For children aged up to one year**, ask whether the child smiles back when the mother smiles at them, or whether they follow the mother with their eyes when she moves around in front of them.
- **For children who can walk**, ask if the child bumps into things or falls over objects on the ground, or has become less mobile.

### What to do

1. Examine the eyes with a torch to check the cornea for any abnormalities, such as haziness or cloudiness associated with congenital glaucoma or corneal ulcers.
2. Perform the red reflex test to check for abnormalities inside the eye, such as cataract or retinoblastoma.
3. If you cannot see any abnormalities, the problem may be inside the eye; for example due to refractive error or conditions affecting the optic nerve or retina. Believe the parent(s) and refer the child **urgently (within one week)** to a hospital with an ophthalmologist.

### What care is needed

The child may have a refractive error and need spectacles, or they may have a form of cerebral visual impairment.

## A child whose eyes are not straight

Before the age of 3 months, the eyes do not work together very well, and may – at times – not appear to look in the same direction at the same time (appear ‘straight’). After the age of three months, the eyes should appear straight most of the time. This should gradually improve until, by six months of age, children's eyes should appear straight all of the time.

### What to do

- Refer **all** children with a squint to an ophthalmologist, as it can be a sign of a serious eye disease, such as retinoblastoma.
- In some communities, a squint is considered attractive, and parents my need more careful counselling. Explain to parents that, if squint is treated too late, the part of the brain responsible for making sense of visual input from that eye will not develop normally, and the child will only be able to see out of their other eye.

### What care is needed

- Children with squint must be examined by an ophthalmologist who can check for, and address, possible causes of squint, such as refractive error, cataract, or retinoblastoma.
- Once these causes are addressed, the squint must be treated. In most children, this is done by patching the ‘good’ eye for periods of time, so that the brain is forced to use the in-turned eye more.
- Surgery and/or spectacles may be needed to correct a squint.

**“In some communities, a squint is considered attractive, and parents my need more careful counselling.”**

## Children and adolescents with red eyes (but no pain)

### Conjunctivitis

In the absence of injury, the most common cause of red eyes in children and adolescents is conjunctivitis, which can be due to infection by bacteria or viruses, or it can be caused by allergies.

If conjunctivitis is due to **bacterial or viral infection**, the commonest symptoms are that the eyes feel sore, gritty and watery, and the eyelashes may be stuck together in the morning.

Distinguishing bacterial infection from viral infection can be difficult.

Bacterial infections are more likely when:

- Only one eye is infected
- There is a lot of discharge, and/or the discharge is yellowish and thick/sticky

Viral infections are likely when:

- Both eyes are usually affected.
- There is some discharge (often clear and watery).
- Several children in the family or school have the same symptoms. This is because some of the viruses responsible can be very infectious.

### What to do

- If it is not clear from the symptoms or history that the child has a viral infection, it is better to err on the side of caution and treat it as a bacterial infection.
- Provide (or prescribe) topical antibiotic eye medication, such as chloramphenicol or gentamicin eye drops, or tetracycline eye ointment.
- Show parents how to instil the drops or apply the ointment.
- Advise parents to do this every 2–3 hours (during the day) to begin with. Depending on the medication and the severity of the child's condition, advise them on how and when to increase the time between treatments, as the infection clears.
- Ask parents to bring the child back in 2–3 days, so you can check whether the infection is resolving.
- Tell parents to visit a hospital immediately if new symptoms such as pain and sensitivity to light develop, as this could suggest their child has a corneal ulcer.
- If the infection does not resolve, refer them to a hospital where there is an ophthalmologist.
- Explain to parents the dangers of using traditional remedies in the eyes.

### Allergic conjunctivitis

There are two broad types of allergic conjunctivitis – one is **acute** (it comes on quickly and usually resolves), and the other is **chronic** (it can last for several years).

In the **acute** form, the eyelids can become very swollen and the eyes become red and very watery. This form of conjunctivitis usually resolves once the person is no longer exposed to whatever caused their allergic reaction (the allergen), but it can sometimes persist.

**Figure 6:**
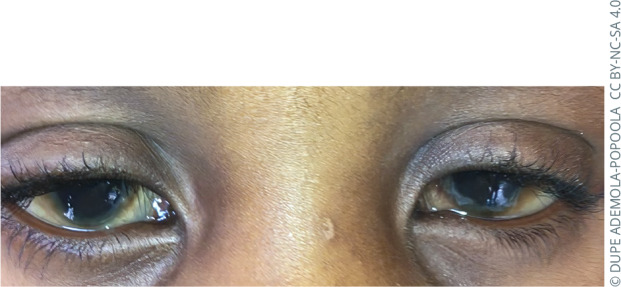
A child with chronic allergic conjunctivities, also known as vernal conjunctivitis. nigeria

The **chronic** form of allergic conjunctivitis, also known as **vernal conjunctivitis**, is more common in children with other allergic conditions such as asthma or eczema. The eyelids are slightly puffy, with a stringy discharge. The eyes can become very sensitive to light. In African and Asian children, the white of the eyes can become brownish (Figure 6).

### What to do

- If a child has **acute allergic conjunctivitis**, offer antihistamine eye drops such as sodium cromoglycate and consider oral antihistamines.
- **Refer** children with **vernal conjunctivitis** to a hospital where there is an ophthalmologist, as they can develop corneal ulcers. Explain to the parents that these children may need long-term treatment and follow-up.

## Eye injuries

Eye injuries are relatively uncommon in preschool-aged children; they increase as children get older.

### Blunt or sharp injuries

**Figure 7:**
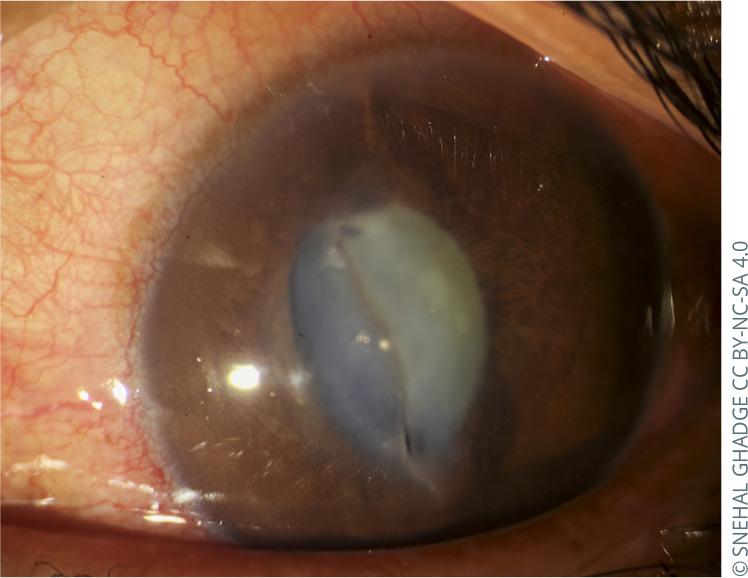
Corneal laceration due to a a sharp object, resulting in damage to the lens. india

Injuries can be blunt, caused by objects such as stones or balls, or sharp, caused by objects such scissors or plant material. Sharp injuries include eye lacerations, such as cuts, tears, or perforations (Figure 7).

### What to do

- Ask the parent or carer what they saw happen, or what they were told happened.
- To be on the safe side, referral is recommended for all eye injuries, as a detailed eye examination is required to see the extent of the injury.

### Chemical burns

**Figure 8:**
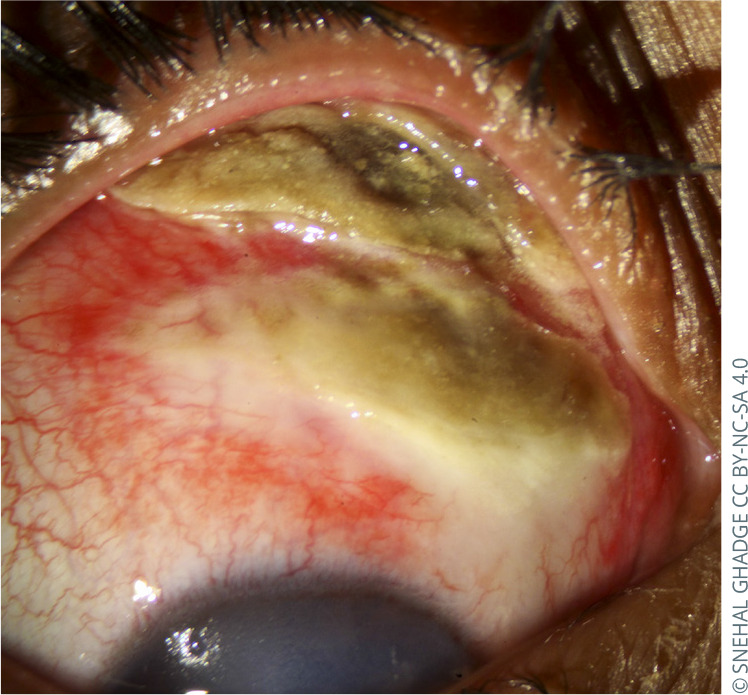
Acute alkali burn. india

Some chemicals can burn the eyes, particularly highly alkaline solutions such as bleach (Figure 8).

### What to do

- If you suspect a chemical entered the eye, first aid is needed. Thorougly irrigate the eyes using sterile water or saline for at least 20 minutes.
- Refer the child **very urgently (on the same day)** to a hospital with an ophthalmologist.

## Older children with poor vision

The most common cause of poor vision in children aged 9–10 years and above is short-sightedness (myopia). The parent or caregiver may notice one or more of the following:

- The child holds things too close to their eyes
- They sit near the screen to watch TV
- They narrow their eyelids when trying to see something.

**“Many common eye conditions in children can be managed at the primary level by either treatment or referral.”**

### What to do

Refer these children for a thorough eye examination, including refraction by an ophthalmologist or optometrist, as they may need spectacles.

In conclusion, many common eye conditions in children can be managed at the primary level by either treatment or referral. Taking a careful history and examining the eyes should give you an indication of what to do. As some conditions are blinding if not diagnosed and managed promptly, refer the child if you are in any doubt. It is important to explain the condition, and how it is treated, very clearly to the carers, and to explain why the referral is urgent, if applicable.

